# Development and reliability of a streetscape observation instrument for international use: MAPS-global

**DOI:** 10.1186/s12966-018-0650-z

**Published:** 2018-02-26

**Authors:** Kelli L. Cain, Carrie M. Geremia, Terry L. Conway, Lawrence D. Frank, James E. Chapman, Eric H. Fox, Anna Timperio, Jenny Veitch, Delfien Van Dyck, Hannah Verhoeven, Rodrigo Reis, Alexandre Augusto, Ester Cerin, Robin R. Mellecker, Ana Queralt, Javier Molina-García, James F. Sallis

**Affiliations:** 1 Department of Family Medicine and Public Health, University of California San Diego, San Diego, CA USA; 2 Health and Community Design Lab, Schools of Population and Public Health and Community and Regional Planning, University of British Columbia, Vancouver, BC Canada; 3 Urban Design 4 Health, Inc., Rochester, NY USA; 4 Institute for Physical Activity & Nutrition, School of Exercise & Nutrition Sciences, Deakin University, Geelong, Australia; 5 Department of Movement and Sports Sciences, Ghent University, Ghent, Belgium; 6 Research Foundation Flanders (FWO), Brussels, Belgium; 7 Prevention Research Center, Brown School, Washington University in St. Louis, St. Louis, USA; 8 Research Group on Physical Activity and Quality of Life, Pontificia Universidade Catolica do Parana, Curitiba, Brazil; 9 School of Public Health, University of Hong Kong, Hong Kong, China; 10 Department of Nursing, University of Valencia, Valencia, Spain; 11 Deparment of Teaching of Musical, Visual and Corporal Expression, University of Valencia, Valencia, Spain; 12 Mary MacKillop Institute for Health Research, Australian Catholic University, Melbourne, Australia

**Keywords:** Exercise, Walking, Built environment, Walkability, Measurement

## Abstract

**Background:**

Relationships between several built environment factors and physical activity and walking behavior are well established, but internationally-comparable built environment measures are lacking. The Microscale Audit of Pedestrian Streetscapes (MAPS)-Global is an observational measure of detailed streetscape features relevant to physical activity that was developed for international use. This study examined the inter-observer reliability of the instrument in five countries.

**Methods:**

MAPS-Global was developed by compiling concepts and items from eight environmental measures relevant to walking and bicycling. Inter-rater reliability data were collected in neighborhoods selected to vary on geographic information system (GIS)-derived macro-level walkability in five countries (Australia, Belgium, Brazil, Hong Kong-China, and Spain). MAPS-Global assessments (*n* = 325) were completed in person along a ≥ 0.25 mile route from a residence toward a non-residential destination, and a commercial block was also rated for each residence (*n* = 82). Two raters in each country rated each route independently. A tiered scoring system was created that summarized items at multiple levels of aggregation, and positive and negative valence scores were created based on the expected effect on physical activity. The intraclass correlation coefficient (ICC) was computed for scales and selected items using one-way random models.

**Results:**

Overall, 86.6% of individual items and single item indicators showed excellent agreement (ICC ≥ 0.75), and 13.4% showed good agreement (ICC = 0.60–0.74). All subscales and overall summary scores showed excellent agreement. Six of 123 items were too rare to compute the ICC. The median ICC for items and scales was 0.92 with a range of 0.50–1.0. Aesthetics and social characteristics showed lower ICCs than other sub-scales, but reliabilities were still in the excellent range (ICC ≥ 0.75).

**Conclusion:**

Evaluation of inter-observer reliability of MAPS-Global across five countries indicated all items and scales had “good” or “excellent” reliability. The results demonstrate that trained observers from multiple countries were able to reliably conduct observations of both residential and commercial areas with the new MAPS-Global instrument. Next steps are to evaluate construct validity in relation to physical activity in multiple countries and gain experience with using MAPS-Global for research and practice applications.

## Background

Relationships between several built environment factors and physical activity are well established [1]. Neighborhood environment features have been classified into two broad categories. Macroscale features include larger, structural and urban form characteristics, such as street connectivity, land use mix, and residential density that are not easily modifiable [2–5]. Microscale features, or smaller details of environments, such as sidewalk or street-crossing quality and aesthetics, are believed to affect people’s confidence, comfort, and safety for walking [6, 7]. In contrast to their macroscale counterparts, microscale features generally can be modified more easily as part of efforts to provide more supportive environments for physical activity.

Numerous observational measures of microscale environments with similar content but different formats have been published and showed good inter-observer reliability [3, 8]. These observational instruments have been developed and used across a wide range of environment types, but they are tailored to local environments. However, we could locate no measures that were designed for international use or evaluated in multiple countries. Physical inactivity is a global health problem that is not improving [9], and built environments have been related to physical activity internationally [10]. Therefore, a common reliable tool designed to capture the diversity of microscale environments found across the globe would foster international comparisons and generate data to inform international initiatives such as United Nations actions to reduce non-communicable diseases [11].

The purpose of the present study was to describe the development and inter-rater reliability of a streetscape observation tool developed for international use and evaluated in several countries. The new measure was based on items, format, and scoring of the Microscale Audit of Pedestrian Streetscapes (MAPS) that was developed in the United States, with several versions shown to be related to physical activity in multiple age groups, including the original 120-item version [6], a 54-item abbreviated version [12], and a 15-item version suitable for use by practitioners [7]. The name of the new measure is MAPS-Global.

## Methods

### Development of MAPS-global

MAPS was originally developed as an observation tool based on prior instruments [8, 13]*.* MAPS has been shown to be a valid [6] and reliable [14] tool for surveying pedestrian environments and microscale urban form features, with some coverage of macroscale attributes such as land use. However, data on the validity and reliability of MAPS were collected in the United States only, and the tool was not designed for international use.

The development of MAPS-Global was part of the International Physical Activity and the Environment Network (IPEN) Adolescent study and led by the IPEN Coordinating Center [15] (www.ipenproject.org). MAPS-Global was intended to be applicable for all ages, from childhood to older adulthood and drew from measures designed for general populations and specific age groups. MAPS-Global was designed to have important physical activity-relevant attributes from every continent in one instrument to allow cross-country comparisons.

To develop a version of MAPS appropriate for global use, the original MAPS and eight additional tools developed for different countries and purposes were identified, and selected items and constructs were adapted to include in MAPS-Global: Bikeability Toolkit (Bicycle Federation of Australia) [16], Assessing Levels of PHysical Activity and fitness (ALPHA; Europe) [17], Environment in Asia Scan Tool (EAST; Hong Kong) [18], Residential Environment Assessment Tool (REAT; UK) [19], Forty Area Study Street View tool (FASTVIEW; UK) [20], Systematic Pedestrian and Cycling Environmental Scan (SPACES; Australia) [21], Sport, Physical activity and Eating behavior: Environmental Determinants in Young people audit tool (SPEEDY; UK) [22], and International Study of Childhood Obesity, Lifestyle and the Environment audit tool (ISCOLE; international) [23]. In addition, a self-report neighborhood environment measure tailored to Africa was considered to enable the use of MAPS-Global in African environments [24, 25]. A document showing the source(s) for each item in the MAPS Global tool can be downloaded at http://sallis.ucsd.edu/measure_maps.html#MAPSGLOBAL [26].

A draft of the MAPS-Global instrument was created through a three-step revision process. First, items from other tools that covered a similar construct as a MAPS item were used to revise the MAPS item to reflect internationally appropriate terms. Second, other modifications were made to existing MAPS items to adapt to international settings, such as increasing the upper range for land uses and building heights. Third, items from all eight instruments were reviewed and considered for inclusion if they met one of the following criteria: the item was found in more than one of the reviewed tools, was considered policy relevant, or captured a feature unique to a region. As most previous tools focused on pedestrian use, special attention was paid to incorporating a bicycling component for MAPS-Global. Table 1 presents a comparison between the original MAPS and MAPS-Global to highlight the changes.

**Table 1:** Summary of changes between the original MAPS and MAPS-Global for each subscale

	Items deleted for MAPS Global (examples)	Items added for MAPS Global	Modifications for MAPS Global
Destinations & Land Use (DLU)			
Positive DLU	Big box store, health/social services, library/museum, post office, senior center, parking facilities, retirement/senior living facility, community garden	Trail, bicycle shop, open air market, bakery	Response scale increased from 2+ to 5+ as upper limit for all land uses except open air markets, strip malls, and shopping centers (shopping mall and shopping arcade combined into 1 item)
Negative DLU	Warehouse, casino, abandoned building, unmaintained lot/field, med-large parking lot	Age-restricted bar/nightclub	None
Items not in subscale	Non-residential buildings with parking lots between walkway and entrance, non-residential buildings adjacent to walkway	Pedestrian street or zone	None
Streetscape Characteristics			
Positive streetscape	Posted speed limit, drainage ditches, drinking fountain, public phones	BRT, train, subway, tram/streetcar, tuktuk/auto rickshaw, bicycle share, hawkers/shops/carts	Modified number of driveways to be collected at the segment level
Negative streetscape	Driveways/alleys, lack of street lights	None	None
Items not in subscale	Senior transit, drainage ditches	None	None
Aesthetics & Social Characteristics			
Positive aesthetics/social	Neighborhood watch signs, commercial signage	Natural bodies of water	None
Negative aesthetics/social	Railroad tracks, extent social disorder, abandoned cars,	Dog fouling	Modified extent of graffiti and litter to include “dog fouling”; combined litter in yards and on street/sidewalk into 1 item
Items not in subscale	Historic/cultural features, Beer/liquor bottles/cans	None	None
Crossings/Intersections			
Positive crossing	Stop lines on road, one-way streets through crossing, audible walk signal, dedicated turn arrows	Crossing on an overpass, underpass or bridge, bicycle signal, tactile paving, bike box	None
Negative crossing	Gutters, steep slope, temporary obstructions, poor visibility at corners	None	None
Items not in subscale	Crosswalk timing, number of legs at intersection, poor condition of crossing surface	None	None
Street Segments			
Positive segment	Building façade colors, building accent colors, building materials	Hawkers or shops, sidewalk or pedestrian street/zone obstructions, pedestrian bridge/overpass/tunnel, covered or air conditioned place to walk along the street or connecting buildings, quality of the bicycle lane/ zone	Modified number of traffic lanes to include pedestrian street; modified coverage of sidewalk/walkway to isolate trees (1 item) and overhangs/awnings (1 item); modified building height to collect shortest (1 item) and tallest (1 item) and upper limit increased from 11+ to 21+ stories; modified bicycle lane/zone with different options; modified street lights to specify for cars (1 item) and for pedestrians (1 item)
Negative segment	Cross-slope, *minor* trip hazards, how much of segment at steepest level	Gates/walls/tall fences around properties	Modified sidewalk or pedestrian zone obstructions to isolate cars; slope estimated by sight rather than measured with an inclinometer
Items not in subscale	Signs to discourage skateboard use, dead-end street	Type of segment: residential or commercial	None
Cul-De-Sacs			
	Diameter of cul-de-sac, slope, condition of pavement, island, parking	Soccer goals, outdoor fitness equipment,	None

After this revision process, a draft of MAPS-Global was distributed to IPEN investigators from 15 countries for review and input. Recommendations for additional items were also solicited during this process. The tool was then finalized for use in the current study and contained 123 items. The tool is available for download [26].

### Study design and cities

Global microscale environmental data were collected for the purpose of this reliability study from November 2014 – June 2015 as part of the IPEN Adolescent study, and included five cities: Melbourne (Australia), Ghent (Belgium), Curitiba (Brazil), Hong Kong (China), and Valencia (Spain). Following IPEN protocol, all cities used a GIS-derived macro-level walkability index to select neighborhoods defined as high versus low walkability, based on: net residential density, intersection density, and mixed land use [27, 28]. Neighborhoods in these cities were selected to represent four neighborhood types categorized as high/low-walkability by high/low-median socio-economic status (SES), to ensure the inclusion of a wide range of demographic and built environment attributes.

The IPEN Adolescent study was approved for research with human subjects by the Institutional Review Boards at Deakin University, Ghent University, Pontifical Catholic University of Parana, University of Hong Kong, and University of Valencia.

### Route selection

To identify routes for MAPS-Global assessment, each country randomly selected 65 IPEN Adolescent study participants, or randomly selected residences within potential study areas, (total *n* = 325) stratified by the four walkability-by-SES neighborhood types. The IPEN Coordinating Center identified each route’s destination as the nearest commercial block. Routes were manually created (0.25–0.45 mile (400–724 m) in distance) from each residence toward a commercial block using Google Earth. The routes were drawn along the road network, providing the most direct route from the residence toward a non-residential destination. Alleys, non-motorized, and informal paths adjacent to the street network were not easily identifiable using online images and were therefore not used to create routes. However, these pedestrian facilities were coded within MAPS-Global when they were observed. MAPS-Global data were also collected along a single road segment at the nearest commercial block to enhance the variety of environmental features assessed, as the 0.25–0.45 mile routes did not always reach the end destination, due to a cap on the maximum surveyed distance (based on time and budget considerations).

### Training

A research staff manager from the IPEN Coordinating Center was responsible for training, route creation, and quality control. Details about length of training and certification can be found elsewhere [14]. Multiple raters at each study site were instructed to use MAPS-Global through an online webinar and were provided training materials including a manual with item definitions and photos (see training manual online [26]). After the online training, each country’s team practiced rating streets in the field and communicated with the IPEN Coordinating Center to clarify site-specific issues. To be certified to rate independently, raters were required to complete observations of at least five routes with inter-rater reliability at 95% agreement or higher.

### Data collection

Data were collected along a 0.25–0.45 mile route (*n* = 325 residential routes) starting at a study participant’s home or a randomly selected residence and walking toward the nearest commercial destination. Data were also collected along 82 commercial blocks. Table 2 describes data collection areas and sample sizes per country.

**Table 2:** Reliability pair sample sizes by country

Country	City	Routes^a^	Segments^bc^	Crossings^c^	Cul-de-sacs
Australia	Melbourne	65	218	108	10
Belgium	Ghent	65	236	156	6
Brazil	Curitiba	65	319	213	0
China	Hong Kong	65	224	145	9
Spain	Valencia	65	350	266	0
Total		325	1347	888	25

^a^residential only, commercial blocks not included

^b^segment defined as the area between crossings

^c^both residential and commercial included

Two raters in each country completed each MAPS-Global route independently. Residential routes took on average 26.1 min to complete (range = 2–100 min) and commercial segments were completed in 15.8 min on average (range = 3–110 min). Raters and coordinators reviewed each tool for missing and discrepant items. If more than 5% of items were missing, raters returned to the route and completed the missing items.

### Scoring and creating subscales

The scoring of MAPS-Global largely followed the original MAPS scoring structure which has been described elsewhere [6, 14]*.* Briefly, the tool has six sections: destinations and land use (DLU), streetscapes, aesthetics and social, street segments (defined as the area between street crossings), street crossings, and cul-de-sacs/dead-ends. DLU, streetscape, and aesthetics/social items were captured at the route level, and these characteristics were generally consistent throughout the route (e.g., speed limit, aesthetics and social environment). Street segment variables, such as sidewalks, buffers between streets and walking spaces, trees, and building setbacks were collected on each segment on the route. Street-crossing variables were measured at every intersection or crossing on the route (e.g., crosswalks, signals). Cul-de-sac variables (e.g., size, amenities) were collected when one or more cul-de-sacs or dead-ends were present within 400 ft (122 m) of the residential address. When multiple segments and crossings occurred along a route, the respective segment and crossing variables were averaged.

This tiered scoring system summarized items into subscales at multiple levels of aggregation. Most sections included positive and negative valence scores based on the expected effect on physical activity. Some items were excluded from subscales due to being transitory (e.g., presence of anyone walking), capturing a particularly important element of the environment (e.g., pedestrian street), or an unclear expected association with physical activity (e.g., segment type). These became single-item indicators.

A modification from the original MAPS was made to adapt to the more destination-dense environments found internationally by increasing the upper range of land use frequency response options to five or more for each type of destination (only “two or more” was used in original MAPS). Land use items were scored as 0, 1, 2, 3, 4, or 5+. Other continuous and descriptive items were dichotomized or trichotomized based on their distributions, theoretical relevance, and compatibility with other scale items’ scoring. In several instances, related items needed to be combined into single variables to be meaningful components of their respective subscales. For example, shortest and tallest building heights were collected as two separate items, but for scoring they were averaged into one variable for the subscale. In such cases, the new variable was computed and then recoded for scoring (e.g., di- or trichotomized) consistently with theoretically related items to match scoring of other items within a subscale.

After items were rescored as necessary, subscale scores were computed by summing the items’ scores. Valence scores were created by summing subscales that were expected to have a positive or negative impact on physical activity based on the consensus of authors familiar with interdisciplinary research, conceptual models, and guidelines. For instance, the sum of the positive destinations and land uses was thought to be positively associated with physical activity, and the presence of social disorder was thought to be negatively associated. All of the positive subscales within a section were summed to create the positive valence score, and the negative subscales were summed for the negative valence score. The streetscape and cul-de-sac sections only contained positively related items. Finally, an overall section score (positive-minus-negative valence scores) was calculated for each main section that contained both of these valence scores. Overall valence scores were calculated by summing the six main sections’ positive and negative scores. The overall grand score was calculated by subtracting overall negative from overall positive scores. The cul-de-sac score was not included in overall valence scores due to an unclear expected association with physical activity.

In addition to section-derived subscales, three new subscales were created from items that were conceptually related but collected within different sections of the tool (e.g., route and segment items). The three new subscales were pedestrian infrastructure, pedestrian design, and bicycle facilities. Detailed information about item recodes, transformations, and subscale creation can be downloaded [26].

### Analysis

The purpose of MAPS-Global was to represent the full international variability in environments, so reliability results were computed on the pooled international dataset. Country-specific reliability estimates would be misleading because different attributes would be rare in each country, leading to reduced variability and low frequency of occurrence of variables that would underestimate reliability. To assess inter-rater reliability, the intraclass correlation coefficient (ICC) was calculated for the MAPS-Global computed scales and several single-item indicators (e.g., place of worship, crossing overpass, etc.). IBM SPSS Version 21 Scale/Reliability procedure was used to compute ICCs using the one-way random model for average measures.

A variety of numeric definitions and adjectival descriptors have been used to classify measures of inter-rater agreement using Cohen’s kappa coefficients for categorical variables and the ICC for test-retest of continuous measures [29–31]. For this study, Cicchetti’s [30] numeric ranges and descriptors were used. The ICC was classified to indicate test-retest reliability that was: ‘excellent’ (ICC ≥ 0.75), ‘good’ (0.60–0.74), ‘fair’ (0.40–0.59), and ‘poor’ (< 0.40). Items with insufficient variability but percentage agreement equal or higher than 75% were considered to have good agreement [21].

## Results

Results presented here were based on pooled analyses for all five study sites. Table 3 summarizes reliability classification levels for individual items that went into scales, single-item indicators, subscales, and overall scores. Using Cicchetti’s criteria [30], 100% of the subscales and overall scores showed “excellent” agreement. Of the 112 individual items and single item indicators for which ICCs or Kappa’s could be computed, 97 (86.6%) had “excellent” reliability, and 15 (13.4%) had “good” reliability. Six of the tool’s 123 items (unanticipated mid-segment crossing, bicycle locker or compound, basketball hoop in cul-de-sac, skateboard feature in cul-de-sac, soccer goal in cul-de-sac, and outdoor fitness equipment in cul-de-sac) were so rare that no ICC or Kappa could be calculated, yet all were retained in the instrument due to their theoretical importance. Two of the “good” agreement individual items (private outdoor recreation and raised crosswalk) and two of the “good” agreement single item indicators (liquor/alcohol store and presence of people walking) had relatively low Kappa’s (0.50–0.59) due to insufficient variability, but had inter-rater agreements from 94.1%–99.9% so were categorized as having “good” agreement [21].

**Table 3:** MAPS-Global ICC/Kappa reliability classifications of individual items, single-item indicators, computed scales, overall scores, and total

		ICC/Kappa Classifications		
		“Excellent” agreement	“Good” agreement	
		(ICCs ≥0.75)	(ICC = 0.60 to 0.74)	
	Median (range)	N (%)	N (%)	Total N (%)
Individual items that make up scales	0.91 (0.50–1.0)	80 (87.0)	12 (13.0)	92^a^ (100.0)
Single-item indicators	0.88 (0.54–0.99)	17 (85.0)	3 (15.0)	20^b^ (100.0)
Subcales (sums of items)	0.94 (0.79–0.99)	21 (100.0)	0 (0.0)	21 (100.0)
Overalls (sums of subscales)	0.96 (0.78–0.99)	13 (100.0)	0 (0.0)	13 (100.0)
Total (items + single item indicators + subscales + overalls)	0.92 (0.50–1.0)	131 (89.7)	15 (10.3)	146 (100.0)

^a^96 total individual items, but 4 items were too rate to compute Kappa’s

^b^22 total single item indicators, but 2 items were too rare to compute Kappa’s

Table 4 provides more detailed results for the key MAPS-Global constructs, including the number of items in subscales, range of scores, items and overall subscale descriptions, and ICC’s/Kappa’s for single-item indicators, subscale, valence, and overall scores. The median ICC was 0.92, with a range of 0.50–1.0. Aesthetics and social characteristics showed lower ICC values than other sections. Liquor/alcohol stores had the lowest ICC, and crosswalk amenities had the highest. The ICC for the overall grand score was 0.99.

**Table 4:** Inter-rater Reliability for MAPS-Global Single Item Indicators, Subscales and Overall Summary Scores

	# items (range of scores)	Sample items and overall subscale description	ICC/Kappa, Confidence Interval
Destinations & Land Use (DLU)			
Positive Destinations & Land Use			
Residential Mix	4 (0–3)	Single family, multi-family, mixed, apartment over retail	.83 (.79, .85)
Shops	8 (0–28)	Grocery, convenience store, bakery, drugstore, other retail, shopping mall, strip mall, open air market	.97 (.96, .97)
Restaurant-Entertainment	4 (0–20)	Fast food, sit-down, café, entertainment	.90 (.88, .92)
Institutional-Service	3 (0–15)	Bank, health-related professional, other service	.94 (.92, .95)
Worship	1 (0–5)	Place of worship	.91 (.89, .92)
School	1 (0–5)	School land use	.98 (.97, .98)
Public Recreation	4 (0–20)	Public indoor, public outdoor facility, park, trail	.83 (.80, .86)
Private Recreation	2 (0–10)	Private indoor, private outdoor facility	.84 (.81, .87)
Pedestrian Street^a^	1 (0–5)	Pedestrian street/zone	.89 (.87, .91)
Negative Destinations & Land Use			
Age-restricted bar or nightclub	1 (0–5)	Age-restricted bar/nightclub	.93 (.91, .94)
Liquor or alcohol store	1 (0–5)	Liquor or alcohol store	.54 (.47, .61)
Positive DLU	27 (0–111)	Sum of the positive DLU subscales	.96 (.95, .97)
Negative DLU	2 (0–10)	Sum of the negative DLU subscales	.92 (.90, .93)
Overall DLU	29^b^	Positive DLU - Negative DLU	.96 (.96, .97)
Streetscape Characteristics			
Transit	9 (0–13)	Number of stops, transit type and amenities (bench, shelter, and timetable)	.90 (.89, .92)
Traffic calming	1 (0–5)	Signs, circles, speed tables, speed humps, curb extension	.90 (.88, .91)
Roll-over curb^a^	1 (0–1)	Roll-over curbs	.84 (.81, .87)
Trash bins	1 (0–1)	Public trash bins	.89 (.87, .91)
Benches	1 (0–1)	Benches or other places to sit	.81 (.78, .84)
Bicycle racks	1 (0–1)	Bicycle racks	.83 (.80, .86)
Bicyle lockers	1 (0–1)	Secure bicycle access lockers or compounds	Too rare to calculate Kappa
Bicycle sharing	1 (0–1)	Bicycle docking station for bike sharing	.97 (.96, .98)
Kiosks	1 (0–1)	Kiosks or information booths	.97 (.96, .98)
Hawkers	1 (0–1)	Hawkers/shops/carts	.97 (.96, .97)
Positive Streetscape	17^c^ (0–25)	Sum of positive streetscape	.93 (.91, .94)
Aesthetics & Social Characteristics			
Presence of anyone walking^a^	1 (0–1)	Presence of anyone walking	.59 (.53, .65)
Positive Aesthetics/Social	4 (0–4)	Hardscape, water, softscape, landscaping	.78 (.74, .82)
Negative Aesthetics/Social	6 (0–6)	Buildings not maintained, graffiti, litter, dog fouling, physical disorder, highway near	.80 (.76, .83)
Overall Aesthetics/Social	10^d^	Positive Aesthetics/Social - Negative Aesthetics/Social	.81 (.77, .84)
Crossings/Intersections			
Positive Crossing Subscales			
Crosswalk Amenities	7 (0–7)	Crossing aids, marked crosswalk, high visibility striping, different material, curb extension, raised crosswalk, refuge islands	.99 (.99, .99)
Curb Quality & Presence	3 (0–6)	Curb presence, curb ramps lined up, tactile paving	.95 (.94, .95)
Intersection Control & Signage	7 (0–7)	Yield signs, stop signs, traffic signal, traffic circle, pedestrian walk signals, push buttons, countdown signal	.97 (.96, .97)
Bicycle Features	3 (0–3)	Waiting area, bike lane crossing the crossing, bike signal	.94 (.93, .95)
Overpass	1 (0–1)	Crossing on pedestrian overpass, bridge	.80 (.77, .82)
Mid-segment crossing^a^	1 (0–1)	Unanticipated mid-segment crossing	Too rare to calculate Kappa
Negative Crossing Subscales			
Road Width	1 (0–2)	Distance of crossing leg	.99 (.98, .99)
Positive Crossing	21 (0–24)	Sum of the positive crossing subscales	.98 (.98, .98)
Negative Crossing	1 (0–2)	Sum of the negative crossing subscales	.99 (.98, .98)
Overall Crossing	22^e^	Positive Crossing - Negative Crossing	.98 (.97, .98)
Street Segments			
Positive Segment Subscales			
Building Height-Setback	4 (0–10)	Building height, smallest and largest setback	.97 (.97, .97)
Segment type^a^	1 (0–1)	Segment type: residential or commercial	.96 (.96, .97)
Building Height-Road Width Ratio	5 (0–3)	Building height, setback and road width	.80 (.78, .82)
Buffer	2 (0–5)	Parking along street, buffer	.92 (.91, .92)
Bike Infrastructure	3 (0–5)	Bike lane presence, quality, signage	.95 (.94, .96)
Shade	3 (0–6)	Number of trees, sidewalk coverage, shade	.93 (.93, .94)
Sidewalk	2 (0–6)	Sidewalk presence and width	.93 (.92, .94)
Pedestrian infrastructure	5 (0–5)	Mid-segment crossing, pedestrian bridge, covered place to walk, street lights	.93 (.92, .93)
Building Aesthetics and Design	1 (0–2)	Street windows	.84 (.82, .85)
Informal Path or Shortcut	1 (0–1)	Informal path connecting to something else	.86 (.84, .87)
Hawkers/Shops	1 (0–2)	Hawkers/shops on sidewalk/ped zone	.73 (.71, .76)
Negative Segment Subscales			
Sidewalk	7 (0–13)	Non-continuous sidewalk, trip hazards, obstructions, cars blocking walkway, slope, gates, driveways	.96 (.95, .96)
Positive Segment	27 (0–45)	Sum of the positive segment subscales	.97 (.97, .98)
Negative Segment	7 (0–13)	Sum of the negative segment subscales	.96 (.95, .96)
Overall Segment	34^f^	Positive Segment - Negative Segment	.98 (.98, .98)
Overall Summary and Grand Scores			
Overall Positive	102 (0–210)	Positive DLU, positive streetscape, positive aesthetics/social, positive segment (mean of all segments), positive crossing (mean of all segments).	.96 (.95, .97)
Overall Negative	16 (0–22)	Negative DLU, negative aesthetics/social, negative segment (mean of all segments), negative crossing (mean of all crossings).	.92 (.90, .93)
Overall Grand Score	118	Overall Positive - Overall Negative	.99 (.99, .99)
Cross-Domain Subscales			
Pedestrian Infrastructure	13 (0–27)	Trail, pedestrian zone, sidewalk presence/width, buffer, shortcut, mid-segment crossing, pedestrian bridge, air conditioned place to walk, low lights, overpass, crosswalk, refuge island	.96 (.95–.97)
Pedestrian Design	13 (0–21)	Open-air market, trash cans, benches, kiosks, hawkers and shops, setback, visibility, pedestrian walk signals, push buttons, countdown signals, ramps, crossing aids	.98 (.97–.98)
Bicycle Facilities	9 (0–11)	Bike racks, docking stations, lockers, bike lane, bike lane quality, signs, bike signal, bike box, bike lane crossing the crossing	.97 (.96–.98)
Cul-de-sacs/Dead-ends			
Overall Cul-de-sac^g^	6 (0–6)	Closeness to participant’s home, total amenities, visibility	.94 (.88, .97)

^a^not included in subscale

^b^31 items in section, bicycle shops added to tool later, pedestrian zone not included in subscales

^c^22 items in this section, 4 new informal transit items added roll over curbs not included in subscales

^d^11 items in this section, presence of people walking not included in subscales

^e^23 items in this section, mid- segment crossing not included in subscales

^f^30 unique items used in subscales, but 5 items (setback × 2, building height × 2, and sidewalk) were scored in more than one way for different subscales, segment type not included in subscales

^g^score reported is based on 2 items as 4 items were too rare to calculate Kappa

## Discussion

To facilitate international comparison of microscale environments relevant to physical activity, a new observational measure (MAPS-Global) was developed by drawing on the previously validated MAPS tool and eight other instruments developed in and for a diverse set of countries. Evaluation of inter-observer reliability of MAPS-Global in five countries indicated all items and scales had “good” or “excellent” agreement. All of the summary scores had “excellent” reliability, with an ICC of > 0.75, and the ICC for the overall grand score was 0.99. The lowest reliabilities for multi-item scales were for the three aesthetics and social characteristics subscales (ICCs = 0.78 to 0.81), though they were still in the “excellent” category. Items dealing with landscaping, water features, dog excrement, and highway nearby may be more difficult to define and require more subjective judgment than other types of items. In general, the results demonstrated that trained observers from multiple countries were able to reliably conduct observations of both residential and commercial areas with the new MAPS-Global instrument.

The development process of MAPS-Global was guided by two considerations. The first was to ensure international applicability by including items relevant to physical activity on every inhabited continent. This was accomplished by including items from environmental measures developed in Africa, the Americas, Asia, Australia, and Europe, as well as adding a bicycling environment subscale. Modifications were also made to existing MAPS items and response scales to capture a wider range of environments. Table 1 summarizes these modifications. IPEN investigators from 15 countries then reviewed, pilot tested, and provided feedback to ensure MAPS-Global would be applicable in their countries. The second consideration was to ensure comparability of measurement across countries. This was accomplished by producing a single instrument supported by a detailed and illustrated instruction manual, delivering training from a central site, and requiring observers to complete an in-field certification process. Although MAPS-Global does not include all possible activity-relevant streetscape features, the included items were deemed most important by consensus of the IPEN Adolescent investigators. Though the instrument was developed as part of a study of adolescents, MAPS-Global was designed to be relevant to all ages.

Strengths of the study were the wide variety of constructs, clear scoring guidelines and training procedures, conceptually meaningful summary variables to use in analyses, and good evidence of inter-observer reliability documented in the present paper. Weaknesses of the measure and the study included the large number of items and need for training and ongoing supervision of observers that add to the costs and investigator burden of data collection. Although MAPS-Global is conceptualized as a measure of microscale attributes, it also includes variables such as land use that can be considered macroscale. The present method of assessing routes from residences toward destinations is not applicable for all purposes, such as evaluating microscale features for an entire neighborhood. However, a protocol has been developed [7] for using MAPS-Global on all or selected street segments by coding “route” items for each segment. Although MAPS-Global was tested in five diverse countries, it has not been examined in low-income countries that may have distinct environmental features or rural areas where MAPS-Global may not be applicable. Future studies using the MAPS-Global tool should include study sites from even more diverse locations, especially low-income countries, to further assess international comparability. Variability in frequency of occurrence of items within countries reduced sample sizes and precluded the presentation of country-specific reliability analyses. Additional refinements may be needed to improve the reliability performance among some of the items that require subjective judgment in future iterations.

## Conclusion

It is important to improve understanding of how cities can be built to support sufficient physical activity and other health indicators [32]. Microscale environment data are lacking internationally, so MAPS-Global promises to fill a critical gap by providing measures of features such as sidewalks, safety of street crossings, and landscaping that are more feasible and affordable to modify than the macroscale layout of cities. Next steps in the evaluation and application of MAPS-Global include examining associations with physical activity (i.e., construct validity), evaluating use of online imagery to facilitate more efficient and cost effective data acquisition, constructing more comprehensive observer training programs, and eventually creating a shorter version of the instrument to encourage more widespread international use. If MAPS-Global is shown to be valid and comparable across countries, it could also be applied to provide evidence for practice and policy, such as identifying strengths and weaknesses of activity-supportive environments within and across cities to inform planning decisions, and evaluating changes in built environments, especially those designed to improve physical activity and health.

## Abbreviations

ALPHA: Assessing Levels of PHysical Activity and fitness
DLU: Destinations and land use
EAST: Environment in Asia Scan Tool
FASTVIEW: Forty Area Study Street View
GIS: Geographic Information Systems
ICC: Intraclass correlations
IPEN: International Physical Activity and the Environment Network
ISCOLE: International Study of Childhood Obesity, Lifestyle and the Environment
MAPS: Microscale Audit of Pedestrian Streetscapes
REAT: Residential Environment Assessment Tool; tool.
SES: Socio-economic status.
SPACES: Systematic Pedestrian and Cycling Environmental Scan.
SPEEDY: Sport, Physical activity and Eating behavior: Environmental Determinants in Young people.
